# COVID-19: it is all about sepsis

**DOI:** 10.2217/fmb-2020-0312

**Published:** 2021-01-25

**Authors:** Jean-Louis Vincent

**Affiliations:** ^1^Department of Intensive Care, Erasme Hospital, Université libre de Bruxelles, 1070 Brussels, Belgium

**Keywords:** acute respiratory distress syndrome, coronavirus, individualization, multiple organ failure, phenotypes

COVID-19, the disease caused by SARS-CoV-2, has affected almost all countries worldwide, stretching hospital and intensive care unit resources to their limits in many areas and causing more than 2 million documented deaths so far. Normal medical and surgical practice has been hugely impacted with many routine procedures canceled and emergency interventions organized according to new, COVID-19-safe protocols to protect staff and patients [1,2].

Initially considered as a community-acquired pneumonia that could worsen into the acute respiratory distress syndrome, it soon became apparent that dysfunction of other organs, in addition to the lungs, is often present in patients with COVID-19; indeed, multiple organ failure (MOF) accounts for most of the deaths from COVID-19 [3]. MOF in COVID-19 can involve any organs, the six most common being the lungs, the cardiovascular system, the kidneys, the brain, the liver and the coagulation system. COVID-19 is characterized by a thrombotic endotheliopathy involving the entire body. The resulting endothelial dysfunction may be the link between COVID-19 and MOF, and the process has been called the cornerstone of organ dysfunction in COVID-19 [4].

Autopsy studies of patients with COVID-19, including one by our group [5], have indicated the presence of viral RNA in all organs. This does not, however, imply that the virus itself is responsible for the alterations in organ function. Indeed, as our understanding of the pathogenesis of COVID-19 has advanced, it soon became clear that the initial phase of viral replication could be followed by an inadequate host response, leading to more generalized disease. By definition COVID-19 is, therefore, sepsis, which is best defined as an inadequate host response to an infection [6]. Importantly, as demonstrated in COVID-19, the causative microorganism in sepsis does not have to be bacterial; sepsis can also be caused by viruses, fungi and even parasites. Mortality rates from severe COVID-19 are similar to those observed in other forms of sepsis [7], and the complex immune response in COVID-19 cannot be distinguished from that of other forms of sepsis.

The immune response to SARS-CoV-2 has been called a ‘cytokine storm’, a term that was initially used to describe the inflammatory cytokine response in the engraftment syndrome of graft versus host disease after allogeneic hematopoietic stem cell transplantation [8]. The term has been challenged in the present setting, because blood cytokine levels are not always very high in these patients, especially compared with levels in patients with non-COVID acute respiratory distress syndrome [9]. Nevertheless, blood cytokine levels are still raised in patients with COVID-19 compared with healthy individuals, and can reach levels similar to those in patients with sepsis due to other causes [10,11]. One should emphasize this is not only a pro-inflammatory response, as blood levels of mediators such as IL-10 and alpha-1 antitrypsin are also elevated [11]. COVID-19 is also associated with increased neutrophil extracellular trap (NET) release and increased levels of free DNA in the blood, and serum from COVID-19 patients triggers release of NETs from control neutrophils [12]. The increased formation of NETs may be involved in the thrombotic endotheliopathy of COVID-19 [13]. Intriguingly, endotoxin levels are often increased in patients with severe COVID-19, and are directly related to the severity of the disease [14]. Obviously, endotoxin cannot originate from the virus, but it may have an endogenous source, released from Gram-negative bacteria of the gut, suggesting the possibility of inadequate blood flow to the various organs, including the gut.

Is using the word sepsis for COVID-19 patients just a question of semantics? No, the implications of calling this sepsis are important. First, this dysregulated host response explains why antiviral therapies, including remdesivir, have not been found to be very effective in COVID-19 patients who have become critically ill. On the other hand, immunomodulation with corticosteroids and tocilizumab are valid therapeutic approaches in some patients, and other options will follow. Second, the recognized importance of thrombotic coagulopathy, reinforced by the common occurrence of pulmonary embolism [15], supports administration of heparin in patients hospitalized with COVID-19. Third, in all patients with sepsis, restoration and maintenance of adequate oxygen delivery to the organs is of paramount importance. The excessive focus on lung edema early in the pandemic initially resulted in liberal use of diuretics, with a secondary decrease in cardiac output and limitation of oxygen delivery. Cardiac involvement can cause severe myocardial depression and pulmonary embolism can cause obstructive shock. Hence, any clinical sign of altered tissue perfusion and/or even a slight increase in blood lactate levels should be an alarm signal to act to improve tissue perfusion. Depending on the individual’s hemodynamic status, this may be achieved by administering intravenous fluid, giving dobutamine as an inotropic agent to increase cardiac output or giving red blood cell transfusions [16].

Recognizing COVID-19 as ‘sepsis’ can also encourage development of therapeutic approaches that target the host response. Unfortunately, too many trials have not attempted to characterize the pathophysiological alterations present in the study patients, yet an excessive host response can be characterized by specific signatures (high C-reactive protein, high ferritin levels, high IL-6 levels, etc.). Increasingly the role of genetic factors in influencing the immune response, including the availability of interferons, is also being highlighted [17,18]. In developing trials for treatments in COVID-19, we should be careful not to repeat the errors made when conducting trials of anti-cytokine treatments in sepsis, in which large heterogeneous populations were included rather than targeting those most likely to respond to the therapy under investigation [19]. Trials of immunomodulating therapies that only enroll COVID-19 patients who have hypoxemia as a unique abnormality are doomed to fail; patients need rather to be characterized according to their immune phenotypes. Instead of attempting to find a ‘one size fits all’ approach to therapy, the time has come to individualize management [20]. Patients with sepsis form a very heterogeneous population and even patients with more specific, COVID-19 sepsis can have multiple characteristics. Careful characterization of these patients can enable the most appropriate treatment to be selected on an individual basis.
